# Impact of female gender and perspectives of pregnancy on admission in residency programs

**DOI:** 10.1186/s12978-018-0559-7

**Published:** 2018-07-05

**Authors:** Elie Attieh, Samer Maalouf, Cynthia Chalfoun, Pamela Abdayem, Elie Nemr, Assaad Kesrouani

**Affiliations:** 1 Obstetrics and Gynecology Department, St Joseph University, Hotel-Dieu de France Hospital, Adib Ishac Street, Achrafie, Beirut, Lebanon; 2 Faculty of Medicine, St Joseph University, Beirut, Lebanon; 3 Oncology Department, St Joseph University, Beirut, Lebanon; 4 Medical Education Department, St Joseph University, Beirut, Lebanon

**Keywords:** Female, Gender, Motherhood, Pregnancy, Program, Residency

## Abstract

**Background:**

Motherhood is a demanding part of any women’s life. Female interns could encounter difficulties during selection for residency program according to their plans of conceiving. Our aim is to explore the influence of female gender on the selection process of residency programs.

**Method:**

A cross sectional study was conducted in 2016 at a University Hospital in Beirut, Lebanon. Female residents and chief of departments were interviewed about the impact of the timing of motherhood during residency on the interview for admission. The questionnaire reviewed concerns among female Lebanese medical residents as well as the head of departments revolving around the choice of opting for motherhood and the decision of integrating into a residency program while juggling motherhood responsibilities.

**Results:**

Eighty nine female residents and 22 head of department agreed to participate in this study. During the interviews for residency acceptance, 29 residents (34.5%) were directly asked about their family and motherhood plans; 9% of them did not reveal their intention. 35% of the residents thought that this subject could affect the program directors’ decision. 47% of residents felt that having pregnant colleagues would add to their workload, and almost half of them (46%) believed that pregnant colleagues showed less productivity. 45% of program directors stated that it was an important factor taken into consideration during the interview, and 68% believed that residents tended to choose their specialty according to their life priorities.

**Conclusion:**

Pregnancy during residency training represents major challenges for female residents and their program directors. Rules and laws designed to set a balance between career and personal life are required to improve women’s ability to participate equally in the workforce.

## Plain Enlish summary

While women accounts for almost half of all medical students, problems encountered due to motherhood in the time of residency still remains an unresolved issue. We investigated the prevalent concerns among female Lebanese medical residents as well as the head of departments revolving around the choice of opting for motherhood and the decision of integrating into a residency program while juggling motherhood responsibilities. A cross sectional observational study on this research problem was conducted at a University Hospital in Beirut, Lebanon. Female residents and chief of departments were interviewed about their point of view regarding motherhood and maternity during residency. There were 89 residents and 22 head of department who agreed to participate in this study. During the interviews, 29 residents (34.5%) were directly asked about their family and motherhood plans, out of which 9% did not reveal their intention. Additionally, 35% of the residents thought that this subject could affect the program directors’ selection of residency candidates. Furthermore, half of them felt that having pregnant colleagues would add to their workload, and believed that pregnant colleagues showed less productivity. 45% of program directors stated that it was an important factor taken into consideration during the interview, and 68% believed that residents tended to choose their specialty according to their life priorities. Pregnancy during residency training represents major challenges for female residents and their program directors. Rules and laws designed to set a balance between career and personal life are required to simplify decision-making in this critical period.

## Background

The existing literature on the subject matter of pregnancy during residency reports that residents struggle with the conflicting identities and roles of a mother, a student, and a physician [1]. The imbalance between having a family life and a medical career was only a minor problem in the past, because the vast majority of doctors were men. In case of women, however, challenges are faced and resolved permanently to achieve this balance.

The number of women pursuing a career in medicine is gradually increasing. Most data comes from North American centers. In 1997, women comprised 43% of the medical students in the United States and 22% of practicing physicians [2]. In 2001, those numbers amounted to 45.8% for female medical students and 28% for female academic medical faculty [3]. Women are expected to represent 50% of all practicing doctors by the year 2040 [4].

Other than their medical profession, women physicians remain responsible for the majority of domestic tasks and the upbringing of their children, making it difficult for them to devote the hours that a profession in medicine necessitates. Women students are hindered by family concerns, while male students are less influenced by them [5].

Pregnancies during residency do occur, and they can cause professional setbacks for many residents [6]. Long hours, unpredictable work demands, guilt induced by the possible workload increase for fellow colleagues, and the residents’ high expectations of themselves can all result in severe stress for pregnant residents [7].

In 2012, a random survey was distributed among 600 female physicians who had graduated from medical school in 1995. Infertility issues occurred in 24% of the respondents, with the average age of diagnosis at 33.7 years. In these cases, when asked what they would have changed in hindsight, 29% stated that they could have considered attempting conception earlier, 17% felt that they should have opted for a different specialty, and 7% wished they had utilized cryopreservation to extend fertility [8].

Another issue frequently encountered by residents concerns the reaction of their colleagues. Fellow residents or practicing physicians may oppose any increase in work that they need to assume in the occasion where pregnant residents reduce their hours of work due to pregnancy or take maternity leaves. Pregnant residents found the physical demands sought by residency and lack of support from fellow residents and their departments to be the most taxing stressors. Feelings of anger and resentment toward the pregnant residents were common among the other residents [6].

This study aims to study the intricate relationship between the personal life and career of female doctors, and the way it impacts the selection into a residency program.

## Methods

A cross sectional study was conducted in 2016 at the Hotel-Dieu de France University Hospital in Beirut, Lebanon, in order to evaluate how maternity and motherhood influence the specialty choice of residents.

A 30-question survey was sent to 143 female residents. A separate 13-question survey was distributed electronically to program directors of all medical and surgical specialties at our University Hospital (a total of 23 departments). There were no exclusion criteria pertaining to residents.

The study protocol was approved by the institution’s ethical committee and the study was registered under the number: tfem/2017/16.

Following an informed consent to participate, the survey was completed anonymously. The consent form was independent from the questionnaire, making the questionnaire anonymous for authors who analyzed the results. Several questions were drafted to address key issues. The survey was pilot tested with a small group; and subsequently, a final check was performed. Residents were asked about their demographics (residency year, social and familial status, the age of the eldest parent). The survey focused on maternity and included questions designed to explore issues surrounding the timing of pregnancy during residency and perceptions regarding the impact of maternity and marriage on the residents’ training as well as on their career and specialty choice.

Head of departments were questioned about their demographics (sex, age, social status), their opinion concerning maternity during residency, and its impact on the resident’s productivity, as well as the impact during the specialty interview.

Questionnaires included closed-end items and open questions for mentioning particular comments. All the questionnaires were submitted over a period of 2 months.

## Results

Among the 143 residents, 89 completed the survey (response rate of 62%). Among the 23 head of departments, 22 completed the survey (95.6%).

The mean age of the residents is 28 years (standard deviation = 1,38 year). Among the 89 residents, only 14 were married (15.7%) and only 5 had kids (35.7% of the married residents). All of them had only one child, and the pregnancy occurred during their third or fourth year. The characteristics of resident respondents and program directors are summarized in Tables 1 and 2 respectively.

**Table 1:** Socio-demographic characteristics of resident respondents

Characteristics	Respondents
Year of residency	PGY1 = 19 (21.3%)
	PGY 2 = 13 (14.6%)
	PGY 3 = 19 (21.3%)
	PGY 4 = 24 (27%)
	PGY 5 = 14 (15.7%)
Marital status	Single = 36 (40.4%)
	In a relationship = 26 (29.2%)
	Engaged = 13 (14.6%)
	Married = 14 (15.7%)
Curriculum status at marriage (*n* = 15)	Internship (6th and 7th year) = 1 (6.7%)
	PGY1 = 4 (26.7%)
	PGY 2, 3 or 4 = 7 (46.7%)
	PGY 5 = 3 (20%)
Number of children (*n* = 5)	1 = 5 (100%)
	2 = 0 (0%)
	> 2 = 0 (0%)
Residency year at first pregnancy (*n* = 5)	Internship (6th and 7th year) = 0 (0%)
	PGY1 = 0 (0%)
	PGY 2, 3 or 4 = 5 (100%)
	PGY 5 = 0 (0%)

**Table 2:** Socio-demographic characteristics of program directors

Characteristics	Respondents
Sex	M = 18 (81.8%)
	F = 4 (18.1%)
Age (years)	40–50 = 3 (13.6%)
	50–60 = 15 (68.1%)
	> 60 = 4 (18.1%)
Social status	Single = 1 (4.5%)
	Married = 21 (95.4%)
Children	Yes = 21 (95.4%)
	No = 1 (4.5%)

With regard to the timing and the opinion of our residents, at the end of their 7th year of medical school, 60/89 (68%) declared that maternity was not an influencing factor for their specialty choice. At the end of PGY1, 63 residents (81%) also declined that maternity was an influencing factor guiding their specialty choice.

During interviews, 29 residents (34.5%) were asked questions pertaining to maternity and parenthood, and most of them (91%) declared that they were honest about their response, while 9% stated that they were not. Indeed, 35% thought that this subject could influence the program director’s selection choice, 24% thought it would not, and 41% were unsure. Conversely, 45% (10 of 22) of program directors revealed that it was an important factor to be taken into consideration during the interview, and 68.1% believed that residents chose their specialty according to their life priorities.

When questioned about their actual priorities, 87.1% of residents confirmed that their education and career came first, and only 12.9% were found to be family-oriented at that stage. Moreover, 63.6% of doctors thought that residents can simultaneously manage both a career and a family. Although 17 program directors (77%) thought that the best time for a resident to have a child was after their subspecialty, 32% of them had their first child during residency.

Despite all the difficulties and obstacles that female residents have to encounter during pregnancy, the motivating factors that lead to their pregnancy include the desire to start a family (85%), encouragement from parents (38%), fear of future infertility (37%), financial independence (16%), and flexible schedules (14%) in that order.

An analysis of the impact of pregnancy on their work revealed that among the 7 residents who have children, during their first trimester, 4 (57%) did not leave work before 6 pm and 2 (29%) stayed back extra hours. During their second trimester, 5 (71%) did not leave work before 6 pm and the other 2 worked extra hours. During their last trimester, 3 (43%) could not stay until 6 pm and had to leave early.

With respect to the point of view of their colleagues, 41 of 88 respondents (46.6%) felt that their pregnant colleagues increased their personal workloads and almost half of them (41/89, 46%) believed that they were less productive.

Program directors thought that marriage alone affects productivity and work (81.8%; *n* = 18) and 91% (*n* = 20) thought motherhood affects their residents’ productivity even further. Details of the effects of motherhood based on these perspectives are summarized in Table 3.

**Table 3:** Response of program directors regarding pregnancy in residency (closed questions, answered by Yes or No)

	Yes **-** *n* (%)
They are a burden to their colleagues	6 (27.2%)
It increases workload for their colleagues	7 (31.8%)
Motherhood affects negatively the quality of work.	7 (31.8%)
Maternity affects negatively academic activities, education and learning	10 (45.4%)
Pregnant residents are treated differently by physicians than their colleagues	11 (50%)
Pregnant residents have less time and energy to care for their children	17 (77.2%)

## Discussion

This is the first descriptive study in Lebanon, the aim of which is to examine residents’ and program directors’ opinions on motherhood during residency.

In our institution, the first specialty decision (deciding between surgical and internal medicine specialties) has to be taken toward the end of medical school, and the second specialty decision takes place at the end of the first year for surgical specialties or the second year for internal medicine specialties. One hypothesis explaining the reasons behind motherhood not being planned before the third year is the simple fact of postponing its stress and effect on career decisions and maybe the program directors’ decision.

In fact, it was found that 34.8% of residents thought that this subject could influence the program director’s choice unfavorably, and 45.4% (10 of 22) of program directors revealed that it was indeed an important factor during the interview.

Trying hard to strike a balance between family life and medical career exerts an impact on future choices (specialty) and education. Choosing the right time to start a family is a crucial decision for women pursuing medicine, especially considering that women’s prime child-bearing years coincide with the peak of her medical studies and residency, when work constraints are considerable and finances are tight, with little disposable income to hire professional assistants (the late 20s and early 30s).

These are popular opinions in many parts of the world—70% of American physicians in their response to a national survey stated that the best time for a pregnancy would be after residency, although 44% of these respondents had been pregnant during residency [9]. A 1996 study revealed that 85% of female physicians make career changes because of their children and families [10]. The pressures presented by increasing age and decreasing fertility are a reality for female physicians. In this study, it was discovered that 36.8% feared future infertility. The dilemna between biological and professional clocks may be a particularly important issue that several female physicians face—one which may have critical implications in terms of professional advancement and potential career satisfaction; despite this fact, delayed child-bearing appears to be common among American female physicians [8]. On an average, female physicians have their first child 7.4 years later than the general population [11]. In this study, the age at which the respondent residents’ first pregnancy occurred was between 28 and 29, which is lower than the average age of 30.4 found in Stentz et al.’s study; Additionally, in this study, 41 of 88 respondents (46.6%) felt that their pregnant colleagues increased their workloads; and almost half of them (46.1%, 41 of 89), believed that they were less productive—all of which correlates with our findings. We note that there is no clear evidence that pregnant residents are less productive (this could be even at the opposite), but we seeked approval or denial of this prevalent opinion among our study population. Lack of support and sometimes hostility from program directors, doctors, and other residents are also extensively reported in the literature [12] [13]. Residents sometimes report receiving less support from colleagues and supervisors upon resuming work post childbirth in a study published in 2005 [7]. Those who had their first pregnancy during medical school received lesser workplace support (68.2%) than those who had their first pregnancies after training (88.6%) [8].

All these problems can be solved by planning for residents’ pregnancies and clear maternity/parental leave policies [6]. Stress reduction can be achieved by opting to live in an area close to work, home, children’s school, and child care. With lesser time spent on traveling between different places or stuck in bottling, more time is available for a productive career and family activities. Managing the increased workloads for colleagues seems to be another challenging aspect of this problem and would impact the decision of faculty administrators regarding the adequate number of residents that would be accepted in a program.

The authors are aware that the results of this pilot study are limited by the number of participants and also by the limited response rate among residents (62%); larger studies involving universities in various countries and societies would be necessary to have a clear view over this important aspect of female physicians.

## Conclusion

The combination of a medical career and motherhood continues to pose challenges for women—a subject matter that warrants further investigation and targeted support. Lebanese training programs can successfully enhance the experience of motherhood during residency by providing policies or laws creating a healthy balance between the competing demands of family, work, and student life. This will certainly have a positive impact on the resident’s productivity and will decrease the extra workload faced by the colleagues of pregnant residents. Authors hope that pointing out this issue will lead authorities in many countries to design targeted rules and laws to set a balance between career and personal life to reinforce women’s ability to participate equally in the workforce during the female residency training period.
